# The Cytosolic Tail of the Golgi Apyrase Ynd1 Mediates E4orf4-Induced Toxicity in *Saccharomyces cerevisiae*

**DOI:** 10.1371/journal.pone.0015539

**Published:** 2010-11-22

**Authors:** Karin Mittelman, Keren Ziv, Tsofnat Maoz, Tamar Kleinberger

**Affiliations:** Department of Molecular Microbiology, Faculty of Medicine, Technion – Israel Institute of Technology, Haifa, Israel; Texas A&M University, United States of America

## Abstract

The adenovirus E4 open reading frame 4 (E4orf4) protein contributes to regulation of the progression of virus infection. When expressed individually, E4orf4 was shown to induce non-classical transformed cell-specific apoptosis in mammalian cells. At least some of the mechanisms underlying E4orf4-induced toxicity are conserved from yeast to mammals, including the requirement for an interaction of E4orf4 with protein phosphatase 2A (PP2A). A genetic screen in yeast revealed that the Golgi apyrase Ynd1 associates with E4orf4 and contributes to E4orf4-induced toxicity, independently of Ynd1 apyrase activity. Ynd1 and PP2A were shown to contribute additively to E4orf4-induced toxicity in yeast, and to interact genetically and physically. A mammalian orthologue of Ynd1 was shown to bind E4orf4 in mammalian cells, confirming the evolutionary conservation of this interaction. Here, we use mutation analysis to identify the cytosolic tail of Ynd1 as the protein domain required for mediation of the E4orf4 toxic signal and for the interaction with E4orf4. We also show that E4orf4 associates with cellular membranes in yeast and is localized at their cytoplasmic face. However, E4orf4 is membrane-associated even in the absence of Ynd1, suggesting that additional membrane proteins may mediate E4orf4 localization. Based on our results and on a previous report describing a collection of Ynd1 protein partners, we propose that the Ynd1 cytoplasmic tail acts as a scaffold, interacting with a multi-protein complex, whose targeting by E4orf4 leads to cell death.

## Introduction

The adenovirus E4orf4 protein is a multifunctional viral regulator. Within the context of the virus, E4orf4 contributes to temporal regulation of the progression of viral infection by down-regulating early viral gene expression [1]–[4], inducing hypophosphorylation of various viral and cellular proteins [4], [5], facilitating alternative splicing of adenovirus mRNAs [5], and regulating protein translation through an interaction with the mTOR pathway [6]. When E4orf4 is expressed individually in transformed cells, it induces p53-independent cell death [7]–[9]. Oncogenic transformation of primary cells in tissue culture sensitizes them to cell killing by E4orf4 [10], indicating that E4orf4 research may have implications for cancer therapy. Induction of cell death by E4orf4 has been reported to be partially caspase-dependent in 293T cells, but it is caspase-independent in other cell lines, suggesting that E4orf4 can induce a non-classical apoptotic pathway, but can also maintain a crosstalk between the non-classical and the caspase-dependent pathways [8], [11]. We have previously shown that E4orf4 interacts with the heterotrimeric protein phosphatase 2A (PP2A) through a direct association with its regulatory Bα/B55 or B′/B56 subunits [2], [12]. The PP2A-E4orf4 interaction mediated by the Bα/B55, but not the B′/B56 subunit is required for induction of cell death [10], [12], [13]. E4orf4 also associates with members of the Src kinase family leading to its Tyr-phosphorylation and to deregulation of Src signaling, resulting in enhanced caspase-independent apoptosis [14], [15]. Analysis of E4orf4 mutants has further indicated that the interactions of E4orf4 with PP2A and Src have a cell-line dependent additive effect on E4orf4-induced cell death [16]. E4orf4 was reported to act both in the nucleus and in the cytoplasm and membranes of mammalian cells [17]–[19]. A key unresolved question is the identity of targets of the PP2A-B55-E4orf4 complex involved in induction of non-classical apoptosis, and the identity of further downstream elements participating in this apoptotic pathway. To further dissect the E4orf4-regulated process, the yeast *S. cerevisiae* was used as a model system. Although yeast cells do not contain all the components of classical apoptotic pathways, they have been previously used as tools for studying human apoptosis-regulating proteins which yielded novel insights into cell death mechanisms [20]. Furthermore, yeast and mammalian PP2A subunits are very similar and thus the E4orf4-PP2A interaction could potentially be conserved in yeast. Indeed, E4orf4 was found to induce PP2A-dependent irreversible growth arrest in yeast [21], [22], indicating that the PP2A-dependent E4orf4-induced cell death pathway is highly conserved in evolution. This high degree of conservation was further confirmed by experiments showing that E4orf4 mutants selected in yeast for loss of their ability to induce toxicity, were also deficient in their ability to induce cell death in mammalian cells [23].

Based on these findings we utilized a genetic screen in yeast, which uncovered a novel component of the E4orf4 cell death-inducing network, called Ynd1. Ynd1 is a Golgi apyrase whose enzymatic activity is required for regulation of nucleotide-sugar import into the Golgi lumen [24], [25]. This protein is inserted in the Golgi membrane, its 500 N-terminal amino acids, including its catalytic domain, are located in the Golgi lumen, whereas its 113 C-terminal residues are found at the cytoplasmic face of the Golgi membrane. The transmembrane domain includes amino acids 501 to 517 [24], [26]. We showed previously that Ynd1 interacted both physically and functionally with Cdc55, the yeast orthologue of the PP2A-B55 regulatory subunit. However, Ynd1 may not be a downstream effector of the E4orf4-Cdc55 complex in yeast, since Cdc55 and Ynd1 deletions confer additive resistance to E4orf4. Moreover, although Ynd1 deletion confers partial resistance to E4orf4 toxicity, overexpression of Cdc55 sensitizes *ynd1Δ* cells to E4orf4 toxicity more efficiently than *WT* cells. E4orf4 was shown to physically interact with Ynd1 in yeast and with the mammalian Ynd1 orthologue, Golgi UDPase, in mammalian cells [27], confirming the evolutionary conservation of this part of the E4orf4 effector network. Surprisingly however, the Ynd1 apyrase activity was shown to be dispensable for mediating E4orf4-induced toxicity [27].

In the present report we investigate which Ynd1 protein domains are required for mediating the E4orf4 signal. We show that the cytosolic tail of Ynd1 is sufficient for efficiently mediating E4orf4-induced toxicity and that it associates with the E4orf4 protein. However, E4orf4 membrane-association does not occur exclusively due to the interaction with Ynd1 since E4orf4 is found in membrane fractions both in the presence and in the absence of this protein. The results presented here will allow us to focus future research more clearly into the mechanisms underlying the contribution of Ynd1 to E4orf4-induced cell death, by searching for proteins associating with the Ynd1 cytoplasmic tail and examining their functional interactions with E4orf4.

## Materials and Methods

### Yeast strains, media, and plasmids

All experiments were carried out in the *ynd1Δ* yeast strain previously described [27]. Yeast cells were grown either in YPD (1% yeast extract, 2% Bacto-peptone, 0.015% L-tryptophan, 2% glucose) or in a synthetic medium containing the appropriate amino acids [28]. For induction with galactose, cells were grown in synthetic medium with 2% raffinose overnight. They were then diluted to OD_600 nm_ = 0.3 and allowed to grow for two more hours before the addition of galactose to 2%. Alternatively, cells were grown in 2% glucose to mid-log phase, washed once in water, and resuspended in 2% galactose.

Plasmids utilized in this work included: B3384 (also known as p424-GAL1), containing a 2 µ origin of replication, a TRP marker and the GAL1 promoter driving the cloned cDNA [29], pGZ418 [24], B3394 (also known as p414-GALL), B3394-E4orf4, pDAD2 and pDAD2-E4orf4 [21]. To generate plasmid B3384-Ynd1, WT Ynd1 containing a 5′-Myc tag and a 3′-HA tag was transferred by BamHI digestion from pGZ148 [24] to plasmid B3384. Ynd1 deletion mutants were constructed as follows: The required region of the cDNA was PCR-amplified from B3384-Ynd1 with primers containing BamHI (5′ primer) or EcoRI (3′ primer) restriction sites. The 5′ primers for N-terminal deletions also encoded a Myc tag, and the 3′ primers for C-terminal deletions encoded an HA tag. PCR-amplified fragments were cloned into plasmid B3384 and verified by sequencing. The primers used for the amplification of the deletion mutants were: 5′ primers: for N-terminal deletions: 371-Ynd1: 5′- CGCGGATCCGTAACCATGGAGCAAAAGCTGATATCTGAAGAGGACTTGAATTGGACGCAAATATTA -3′; 468-Ynd1: 5′- CGCGGATCCGTAACCATGGAGCAAAAGCTGATATCTGA

AGAGGACTTGAGTGAAAGAAGAACTAAG -3′; Ynd1-518: 5′- CGCGGATCCGTAACCATGGAGCAAAAGCTGATATCTGA

AGAGGACTTGCATAGGTCACACATAATC -3′; 3′ primers: for N-terminal deletions: 371-Ynd1, 468-Ynd1, Ynd1-518: 5′- GGAATTCCTCAGGCGTAGTCCGGGACGTCATATGG -3′; for C-terminal deletions: 371-Ynd1-531, 468-Ynd1-531: 5′- GGAATTCCTCAGGCGTAGTCCGGGACGTCATATGGGTA

GTACAGACCGGAAAAACG -3′.

### Random Mutagenesis of Ynd1

Random mutagenesis of Ynd1 was performed using the GeneMorph II EZClone Domain Mutagenesis Kit by Stratagene, according to the manufacturer's instructions.

### A yeast Serial Dilution Assay

A starter of yeast cells was grown overnight at 30°C in the appropriate liquid medium containing glucose as a carbon source. Cells were diluted to OD_600 nm_ = 0.1 and were then serially diluted five more times at a 1∶5 ratio. 3 µl of each dilution were plated on the appropriate selection plates containing either glucose or galactose as a carbon source. The plates were incubated at 30°C for 3–5 days.

### A quantitative comparison of the mediation of E4orf4-induced toxicity by the various Ynd1 mutants

*ynd1Δ* cells transformed with wild-type (WT) Ynd1, an empty vector or each of the mutants, as well as E4orf4 or an empty vector, were grown overnight in liquid medium containing glucose as a carbon source. The yeast suspension was then diluted to OD_600 nm_ = 0.05 and duplicate aliquots of each sample were plated on the appropriate selection plates containing either galactose as a carbon source or glucose, serving as a plating control. After six days of growth, colonies were scanned, and the total area of the colonies from each sample was measured using the Image Pro Plus 5 software. To compare between the size of colonies containing an empty vector and the size of colonies expressing E4orf4 for each Ynd1 mutant, the ratio between total colony area in the absence and presence of E4orf4 was calculated. This ratio was normalized to the parallel ratio of colony areas on glucose plates. This normalization abolished differences created by fluctuations in yeast concentrations in the various samples. The ratio obtained was further normalized to the ratio calculated for the WT Ynd1 protein, and the final values provide a relative quantitative measure of the toxic effect of E4orf4 on yeast growth in the presence of WT or mutant Ynd1 constructs. Since colonies containing an empty vector are larger than colonies expressing E4orf4, a higher colony size ratio represents a higher ability of the Ynd1 construct to mediate E4orf4 toxicity.

### Immunoblot analysis and coimmunoprecipitation of yeast proteins

For immunoblots and coimmunoprecipitation experiments, yeast extracts were prepared by bead-beating cells for 4 min at 4°C in lysis buffer (250 mM NaCl, 50 mM Tris-HCl, pH 7.4, 5 mM EDTA, 0.5% Nonidet P-40, 0.1% Triton X-100, 50 mM NaF, 100 µM sodium vanadate, 100 nM okadaic acid (Calbiochem), 1× complete protease inhibitor mixture (Roche Applied Science), 1∶250 Sigma protease inhibitor mixture for use in yeast extracts, and 2 µg/ml aprotinin (Sigma)). The lysates were spun to separate beads and debris from the clear lysate. The beads were washed twice more in the lysis buffer, and immunoprecipitations were carried out in the same lysis buffer. Antibodies used in this work included: anti-Myc (9E10) and anti-HA (Covance, Berkeley, CA), anti-E4orf4 [7], anti-Tpd3p (from J. R. Broach), and anti-Rbp4 (Neoclone, Madison, WI).

### Subcellular Fractionation of Yeast

Yeast cells were lysed by bead beating for 4 minutes at 4°C in homogenization buffer (50 mM Tris-HCl pH 7.5, 0.3 M sucrose, 5 mM EDTA, 1 mM EGTA, 5 mg/ml BSA, 2 mM DTT, protease inhibitors as above). Lysates were centrifuged at 1,000 g for 5 minutes to remove nuclei and partially lysed cells. The supernatants were centrifuged at 10,000 g for 10 minutes to pellet the heavy membrane fraction. The resulting supernatants were centrifuged at 120,000 g for 1 hour. The pellets of this centrifugation step contained the light membrane fraction, and the supernatants represented the cytosolic fraction. All centrifugation steps were done at 4°C. Most of the ER, vacuolar membranes and plasma membrane are found in the heavy membrane fraction, whereas the light membrane fraction is enriched in Golgi membranes [24].

### Proteinase K protection assay

Crude membrane extracts were prepared as above, except for omitting the centrifugation step at 10,000 g. The pellet containing total cellular membranes was suspended in Proteinase K buffer (10 mM Triethanolamine pH 7.2, 0.8 M Sorbitol, 1 mM EDTA) and subsequently treated with one of the following: A) Proteinase K buffer; B) Proteinase K buffer +1% Triton X-100; C) Proteinase K buffer +200 µg/ml Proteinase K (New England BioLabs, Ipswitch, MA); D) Proteinase K buffer +1% Triton X-100+200 µg/ml Proteinase K. All reactions were incubated at 37°C for 45 minutes, and were stopped by addition of 100% TCA to the sample. The samples were centrifuged at 18,000 g for 10 minutes at 4°C. The supernatants were discarded, and the pellets were washed with cold acetone. Dried pellets were suspended in protein sample buffer and separated by SDS-PAGE.

### Image Acquisition and Processing

Yeast colonies were photographed with the Bio-Rad ChemiDoc™ documentation apparatus or scanned using an Epson Perfection 4990 scanner. Blots were scanned using the same scanner. Images were processed using Adobe PhotoShop 5.0 or 7.0 and colony areas were quantified using the Image Pro Plus 5 software.

## Results

### Random mutagenesis of Ynd1

To characterize the role of Ynd1 in the signal transduction pathway induced by E4orf4 and to map Ynd1 protein domains that are essential for mediation of E4orf4-induced toxicity, a random mutagenesis approach was initially taken. Plasmids containing randomly mutagenized Ynd1 DNA were transformed into *ynd1Δ* yeast cells expressing E4orf4 and viable colonies were selected on galactose plates (both Ynd1 and E4orf4 were expressed from a galactose-inducible promoter). In parallel, control yeast samples were transformed with empty vector and E4orf4, or with WT Ynd1 and E4orf4. The sizes of colonies obtained in the screen were compared to sizes of control colonies. Colonies which grew similarly to those obtained by transformation with E4orf4 in the absence of Ynd1 were expected to express Ynd1 mutants which were unable to mediate the E4orf4 signal, and may thus contain biologically-significant mutations. Out of approximately 5,000 transformants growing on glucose plates, 200 normal-sized colonies grew on galactose plates. Ynd1 expression in these colonies was assessed using Western Blot analysis. Six of the colonies expressed wild-type levels of truncated forms of Ynd1. The other colonies expressed either very low levels of Ynd1 or no Ynd1 at all (results not shown). Plasmid DNAs extracted from the yeast colonies expressing WT levels of Ynd1 variants were sequenced. Sequences of all 6 mutants which were expressed at wild-type levels revealed a nonsense mutation creating a premature stop codon. The stop codons replaced codons for amino acids at positions 136, 243, 318, 356, 360 and 419. Thus, this approach yielded only mutants expressing truncated Ynd1 proteins containing the N-terminal part of the protein, which is normally localized to the Golgi lumen [24]. All truncated proteins lacked the transmembrane domain of Ynd1. These findings suggested either that the N-terminal part of the protein must be inserted in the Golgi lumen and therefore the transmembrane domain should be retained to facilitate signal transduction, or that the C-terminal cytosolic domain of the protein was required for mediation of the E4orf4 toxic signal.

### Mediation of E4orf4-induced toxicity by Ynd1 truncation mutants

**Figure 1:**
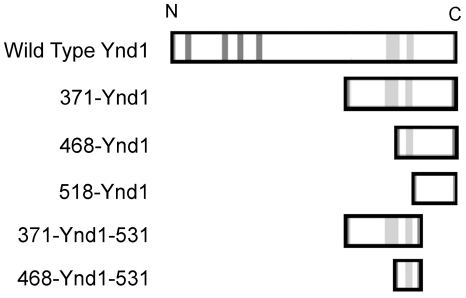
Ynd1 constructs. A schematic representation of WT and mutant Ynd1 proteins is shown. The dark grey lines in the WT protein represent apyrase-conserved-domains found in the N-terminal, luminal part of the protein, and the light grey lines represent hydrophobic domains. The rightmost hydrophobic domain was determined to be the transmembrane domain [26]. All Ynd1 constructs carry a Myc tag at their amino terminus (N) and a HA tag at the carboxyl terminus (C).

To further investigate which domain of Ynd1 was required for the mediation of E4orf4-induced toxicity, three Ynd1 mutants containing N-terminal deletions were generated. Mutant 371-Ynd1 encodes Ynd1 with an N-terminal deletion of amino acids 1–370 of the WT protein, 468-Ynd1 encodes Ynd1 with an N-terminal deletion of amino acids 1–467 of the WT protein, and mutant 518-Ynd1 contains only the cytosolic tail of Ynd1 (amino acids 518–630), lacking the transmembrane domain. In addition, mutants lacking both N-terminal 370 or 467 residues and the C-terminal 99 amino acids were generated (371-Ynd1-531 and 468-Ynd1-531 respectively). The WT protein as well as all mutants contained an N-terminal Myc-tag and a C-terminal HA-tag and were expressed from a galactose-inducible promoter. The schematic structure of the mutants and the wild-type protein can be seen in Fig. 1.

**Figure 2:**
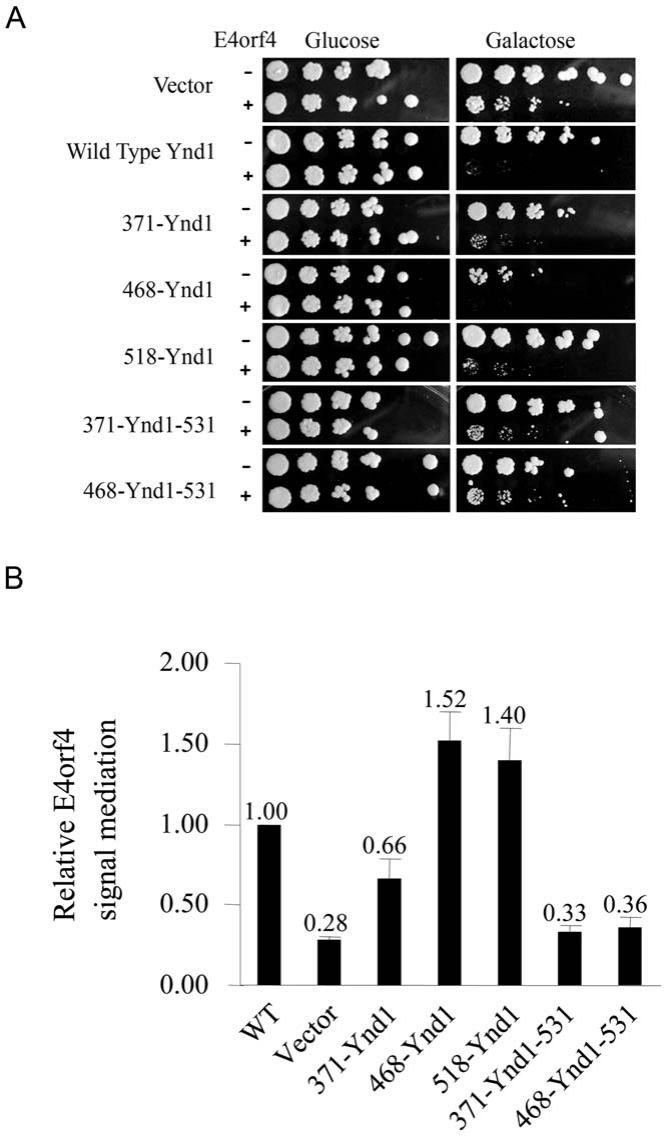
Transduction of E4orf4 toxicity by WT and mutant Ynd1 constructs. **A**. *ynd1Δ* cells transformed with WT Ynd1 or mutant Ynd1 constructs in the presence or absence of E4orf4 were serially diluted (1∶5) and grown on glucose and galactose plates. **B**. *ynd1Δ* cells were transformed with WT Ynd1 or mutant constructs in the presence or absence of E4orf4. Yeast cell concentrations were equalized according to OD measurements, and equal amounts of yeast were grown on glucose and galactose plates. A quantitative comparison of the ability of WT and mutant Ynd1 constructs to mediate the E4orf4 toxic signal was performed as described in the Materials and Methods section, and reflects relative colony sizes in the presence and absence of E4orf4. The activity of WT Ynd1 was defined as 1. Three experiments were carried out containing 2 duplicates each. Error bars represent the standard error.

To test which Ynd1 mutants mediated the toxic effect of E4orf4, the mutant constructs were introduced into *ynd1Δ* yeast cells expressing E4orf4 from a galactose-inducible promoter or into control *ynd1Δ* cells containing an empty vector and a serial dilution assay was performed (Fig. 2A). *ynd1Δ* cells expressing WT Ynd1 or an empty vector served as controls. It should be noted that lack of *YND1* conferred only partial resistance to E4orf4 toxicity, indicated by the smaller colony sizes in *ynd1Δ* cells expressing E4orf4 in comparison to *ynd1Δ* cells containing vector alone (Fig. 2A, vector). However, colony sizes were further reduced when WT Ynd1 was added to the cells ([27] and Fig. 2). When comparing sizes of colonies obtained by transformation of *ynd1Δ* cells with E4orf4 and the various Ynd1 deletion mutants, it appeared that the three N-terminal deletion mutants could transduce the E4orf4 signal at least partially, whereas the short 371-Ynd1-531 and 468-Ynd1-531 mutants did not efficiently transduce the signal (compare the sizes of individual colonies). However, using this assay it was impossible to quantify the relative performance of the mutants, since they also had varying effects on yeast growth in the absence of E4orf4. For this reason, a quantitative colony size comparison assay was designed, which took into account the differential effects of the various Ynd1 mutants on yeast growth in the absence and presence of E4orf4 (described in detail in the materials and methods section). *ynd1Δ* cells were transformed with a plasmid expressing WT Ynd1, an empty vector, or one of the mutants, together with an E4orf4-expressing plasmid or its corresponding empty vector. The transformants were grown overnight at 30°C in liquid medium containing glucose as a carbon source, in which neither E4orf4 nor the Ynd1 constructs were expressed, and were then diluted and plated for the quantitative assay. Colony size ratios calculated for each of the mutants, as well as the ratios for WT Ynd1 and the empty vector control can be seen in Fig. 2B. The results demonstrate that mutant 371-Ynd1 mediated E4orf4-induced growth arrest less well than wild-type Ynd1 (66% of WT) but E4orf4-induced toxicity was higher in its presence than it was when Ynd1 was absent (vector, 28% of WT). Mutant 468-Ynd1 mediated the E4orf4 toxic signal 1.5-fold better than the WT protein. Compared with the 371-Ynd1 mutant, 468-Ynd1 lacks a hydrophobic domain adjacent to the transmembrane domain, which may affect the topology and activity of Ynd1 protein domains both inside and outside the Golgi. Surprisingly, mutant 518-Ynd1, containing only the cytosolic tail of Ynd1, was 1.4-fold more efficient than the WT protein in mediating the E4orf4 signal. In contrast, the short Ynd1 mutants (371-Ynd1-531 and 468-Ynd1-531), lacking most of the cytosolic tail in addition to their N-terminal deletions, did not transduce the E4orf4 signal significantly better than the empty vector. These results suggest that the cytosolic tail of Ynd1 mediates E4orf4 toxicity even in the absence of the luminal and transmembrane domains of the protein. It should be noted that a similar effect of the 518-Ynd1 mutant was observed using non-tagged 518-Ynd1 and WT proteins (not shown), indicating that the tags did not have an independent effect in this system.

### Membrane localization of Ynd1 proteins and E4orf4

**Figure 3:**
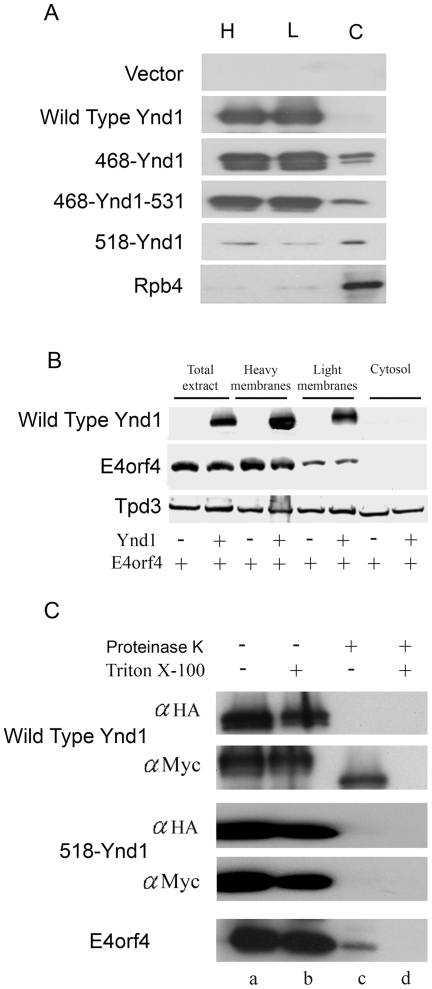
Subcellular localization of Ynd1 proteins and E4orf4. **A**. *ynd1Δ* yeast cells expressing the indicated Ynd1 constructs were subjected to subcellular fractionation by differential centrifugation, and the presence of Ynd1 proteins in the various fractions was determined by a Western blot analysis. Rpb4 is a RNA polymerase II subunit which shuttles between the nucleus and the cytosol, and served here as a cytosolic fractionation marker. H: heavy membranes. L: light membranes. C: cytosol. **B**. *ynd1Δ* yeast cell extracts expressing vector control or WT Ynd1 together with E4orf4 were subjected to subcellular fractionation by differential centrifugation. Western blots were stained sequentially with antibodies recognizing tagged Ynd1, E4orf4, and the Tpd3 subunit of PP2A. **C**. Crude membranes were prepared from yeast cells expressing the indicated Ynd1 constructs or E4orf4, and were subjected to a Proteinase K protection assay in the presence or absence of Triton X-100. Proteins were analyzed by Western blots stained with the indicated antibodies. The Myc tag is fused to the amino terminus of Ynd1 constructs and the HA tag is fused to the cytosolic carboxyl tail. In this experiment, a longer exposure of the 518-Ynd1-containing blot allowed the presentation of a stronger signal in the blot to confirm that most of the protein was degraded by Proteinase K.

**Figure 4:**
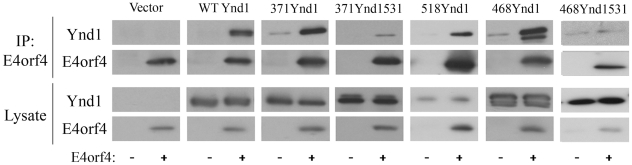
Coimmunoprecipitation of E4orf4 with WT and mutant Ynd1 proteins. E4orf4 was immunoprecipitated from *ynd1Δ* yeast cells expressing the indicated Ynd1 proteins. Western blots of the immune complexes (IP) and input lysates (10% of IP) were stained with the indicated antibodies.

The differences in the ability of Ynd1 mutants to mediate E4orf4-induced toxicity could possibly reflect altered subcellular localizations. To examine whether Ynd1 mutants remained membrane-associated, light and heavy membranes were separated from cytosol and nuclei by differential centrifugation, and the presence of WT and mutant Ynd1 proteins in the various fractions was compared by a Western blot. It has been previously reported that most of the ER, vacuolar membranes and plasma membrane are found in the heavy membrane fraction, whereas the light membrane fraction is enriched in Golgi membranes [24]. As seen in Fig. 3A, WT Ynd1 could only be detected in the membrane fractions (both heavy and light), whereas a small percentage of mutant proteins 468-Ynd1 and 468-Ynd1-531 could be found in the cytosol. However, since this minor cytosolic presence of the mutants did not correlate with their efficiency in mediating the E4orf4 signal (Fig. 2), it may not be significant. The finding that mutant 468-Ynd1 appears as two bands, whereas the other mutants and the WT Ynd1 proteins appear as a single protein band (Figs. 3, 4) may result from more pronounced changes in glycosylation of this specific mutant. Mutant 518-Ynd1 was observed to be partially cytosolic, but densitometric analysis of the blot revealed that 50% of this protein remained membrane-associated. In contrast, 90% of the RNA polymerase II Rpb4 subunit, which was reported to localize both to nuclei and cytoplasm, but not to membranes [30], was found in the cytosol. These results indicate that the subcellular fractionation was efficient and that indeed mutant 518-Ynd1 was partially membrane-associated despite lacking the transmembrane domain.

Since the cytosolic tail of Ynd1 was partially membrane-associated and appeared to mediate the E4orf4 signal, we examined whether E4orf4 was also associated with the cytosolic face of membranes. First we used differential centrifugation to determine whether E4orf4 was membrane-associated. Fig. 3B demonstrates that E4orf4 co-purified with both light and heavy membrane fractions, but was not found freely in the cytoplasm, whereas the PP2A-A/Tpd3 subunit was found both in membranes and in the cytoplasm. Interestingly, membrane localization of E4orf4 was not dependent on the presence of Ynd1, since the association of the viral protein with membranes was similar in the presence or absence of Ynd1. To confirm that E4orf4 localized to the cytoplasmic face of membranes, a protease digestion protection assay was used. This assay is based on the fact that cytoplasmic-facing domains of organelle membrane proteins can be digested by proteases in intact membranes in the absence of membrane solubilizing agents such as Triton X-100, whereas protein domains that are not accessible to the proteases can only be digested after membrane solubilization. Crude membrane fractions were isolated and subjected to Proteinase K digestion in the absence or presence of Triton X-100, as previously described [24]. Fig. 3C demonstrates that Proteinase K digestion of intact membranes (in the absence of Triton X-100) caused an almost complete disappearance of E4orf4, indicating that the majority of this protein was found at the cytosolic face of the membrane, accessible to protease digestion. At the same time, only the cytosolic tail of Ynd1 was degraded by Proteinase K in the absence of Triton X-100, as determined by detection of the amino terminal Myc tag fused to Ynd1, and as was previously reported by Zhong and Guidotti [24]. The 518-Ynd1 cytosolic mutant was completely digested by the protease in the absence of Triton X-100, confirming the conclusion that it associated with the Golgi apparatus at its cytosolic face.

### Coimmunoprecipitation of Ynd1 mutants with E4orf4

We have previously shown that Ynd1 associates with E4orf4 [27]. To investigate whether Ynd1 mutants mediating the E4orf4 toxic signal, and specifically the cytosolic mutant, have retained the ability to interact with E4orf4, coimmunoprecipitation experiments were carried out. Fig. 4 demonstrates that WT and several mutant Ynd1 proteins were coimmunoprecipitated with E4orf4. However, only low levels of the short non-functional Ynd1 mutants were found in E4orf4 immune complexes, compared with the functional Ynd1 proteins (compare for example 371-Ynd1 and 371-Ynd1-531). The cytosolic 518-Ynd1 mutant was expressed at relatively low levels, but could still be efficiently coimmunoprecipitated with E4orf4. Only very low non-specific binding of Ynd1 to the E4orf4-specific antibodies was observed in the absence of E4orf4. Thus, the Ynd1 cytosolic tail can associate specifically with E4orf4.

## Discussion

E4orf4-induced toxicity in yeast has been previously shown to require the Cdc55 subunit of PP2A and the Golgi apyrase Ynd1. Both proteins contribute additively to E4orf4 toxicity, and interact physically and functionally [27]. Ynd1 contains an active nucleoside hydrolysis site located in the Golgi lumen, a transmembrane domain, and a cytosolic tail [24], [26]. In this work we identified the Ynd1 cytosolic tail as a Ynd1 domain required for transduction of the E4orf4 signal. We have shown that this domain, when detached from the rest of the protein, acts as a membrane-associated protein (Fig. 3), can mediate the E4orf4 signal (Fig. 2), and associates with the E4orf4 protein (Fig. 4). These findings are consistent with previous results showing that the luminal apyrase activity is not required for mediation of the E4orf4 signal [27]. Although E4orf4 is found at the cytosolic face of yeast membranes, as judged by cell fractionation and Proteinase K protection assays (Fig. 3), membrane localization of the viral protein is not dependent exclusively on the presence of Ynd1, since it was found to be membrane-associated in *ynd1Δ* cells (Fig. 3B). Based on proteomic prediction tools (Sosui, DAS, TMPRED, TOPPRED), E4orf4 may contain a hydrophobic patch that creates an amphipathic helix. Thus it may be recruited to cell membranes either through its hydrophobic region or by interacting with other membrane-associated proteins. E4orf4 and Ynd1 were found both in light and heavy membrane fractions, suggesting that they associate with both the Golgi apparatus and additional cell membrane compartments. Indeed, mammalian Ynd1 was previously found in lysosomal/autophagic vacuole membranes [31], as well as in the Golgi (our unpublished results).

The cytosolic 518-Ynd1 mutant was usually expressed at lower levels than the other mutants (Fig. 4), but transduced the E4orf4 toxic signal at least as well as these mutants, if not better (Fig. 2). It is possible that the flexibility of the cytoplasmic tail is dictated, at least in part, by the mechanism by which this domain is anchored to the membrane. It has been previously suggested that most of the COOH-terminal cytosolic tail of Ynd1 may bind to the cytoplasmic face of Golgi membranes because of its highly positive charge [24]. The absence of additional membrane anchoring through the transmembrane domain may increase the flexibility of the peripherally-anchored tail and enhance its accessibility to interacting proteins.

Our results and previous reports on Ynd1-associating proteins suggest the possibility that the Ynd1 cytosolic tail mediates E4orf4 toxicity by acting as a scaffold for a multi-protein complex which is targeted by E4orf4. We have previously shown that E4orf4 dissociates Cdc55 from Ynd1 [27], however this finding could represent the disruption of a multi-protein complex which includes Cdc55 and other proteins as well. The *Saccharomyces* Genome Database cites at least 10 membrane proteins which physically associate with Ynd1, as determined by a two-hybrid screen and by affinity capture experiments [32], [33]. Six of these proteins, Erd1, Erv29, Pmp2, Sys1, Yip3 and a putative YPL264C protein, were found in the BioGRID database to interact with each other, and they could thus be part of a large protein complex, which includes Ynd1. Interestingly, the yeast interaction screen used to identify these proteins, the “split-ubiquitin membrane yeast two-hybrid system”, was designed in such a way that it favored the identification of interactions between carboxyl termini of integral membrane proteins which are localized at the cytoplasmic faces of cellular membranes [32]. As a result, it is likely that Ynd1-associating proteins identified by this method interact with the Ynd1 cytosolic tail, and are thus also accessible for interaction with E4orf4. Moreover, since this interaction screen was designed to reveal interactions between integral membrane proteins, it could not reveal cytosolic proteins that may be recruited to membranes, such as Cdc55. Thus it is possible that the Ynd1 cytosolic tail recruits additional peripheral membrane or cytosolic proteins. Since E4orf4 can associate with membranes in the absence of Ynd1 (Fig. 3B), it may interact with other members of a Ynd1 multi-protein complex. This interaction could possibly lead to dissociation of the complex and to further transduction of the E4orf4 toxic signal. Alternatively, E4orf4 could bind both the Ynd1 cytosolic tail and additional membrane proteins and bring them together.

Four of the six Ynd1-associating proteins which are potential members of a multi-protein complex are involved in ER to Golgi vesicle-mediated transport (Erv29 and Yip3) and vesicle organization (Sys1) or ER retention of cargo proteins (Erd1). Two of these proteins, Yip3 and Sys1, have also been implicated in endosomal trafficking. Three other Ynd1-interacting proteins, which were not found to interact with other Ynd1-binding proteins, were classified as vacuole-associated proteins. Thus it appears that Ynd1 associates with several components of both early and late secretory pathways in yeast, and it is possible that E4orf4 interacts with a secretory protein complex anchored to the Ynd1 cytosolic tail.

It has been recently reported that in mammalian cells, E4orf4 promotes transport of recycling endosomes to the Golgi apparatus and inhibits recycling of protein cargos to the plasma membrane through its regulation of Src family kinases, Cdc42, actin dynamics, and Rab11a [34]. These events lead to Golgi membrane scattering and contribute to cell death. Although yeast cells lack Src family members, it is possible that E4orf4 may also utilize a backup mechanism which allows it to interact directly with components of the secretory pathway to affect protein trafficking, thus further transducing its toxic signal. It is not currently known whether early or late steps in protein sorting may be affected by direct interaction with E4orf4.

It has been previously reported that the E4orf4-PP2A complex interacts with the anaphase-promoting complex/cyclosome (APC/C) and modulates its activity at both G2/M and S phases of the cell cycle, thus contributing to E4orf4-induced toxicity [21], [35]. Furthermore, we have shown that Ynd1 interacts genetically with both the Cdc20 and Cdh1/Hct1 activating subunits of the APC/C [27]. These results indicate that PP2A, Ynd1 and the APC/C are all part of the E4orf4 network inducing toxicity. These three effectors could interact either at the cytoplasmic face of the Golgi, or at other locations, such as spindle poles which were reported to co-localize not only with the APC/C [36], but also with regulators of endocytic traffic, such as clathrin and rab11 [37], [38]. Future identification of Ynd1-containing complexes in the presence and absence of E4orf4 will provide more information on their contribution to E4orf4-induced toxicity.

In summary, we propose that the Ynd1 cytosolic tail may act as a scaffolding domain allowing the association of E4orf4 with Ynd1 and additional Ynd1 partners, and this interaction contributes to transduction of the E4orf4 toxic signal.
